# Older Physical Education Teachers’ Wellbeing at Work and Its Challenges

**DOI:** 10.3390/ijerph192114250

**Published:** 2022-10-31

**Authors:** Henry Lipponen, Mirja Hirvensalo, Kasper Salin

**Affiliations:** Faculty of Sport & Health Sciences, University of Jyväskylä, 40014 Jyvaskylan, Finland

**Keywords:** physical education, older employee, ageing, work ability, coping at work, wellbeing at work

## Abstract

This article examines older physical education (PE) teachers’ wellbeing over the course of their career in Finland. The study highlights challenges to physical and mental functioning as well as how teachers respond to these challenges. The six interviewees were over 55-year-old PE teachers, whose career had lasted for more than 30 years. Qualitative methods were used in the collection, transcription and analysis of the research data. The qualitative analysis consisted of a series of interpretations that visualised the world described by the interviewees. All the research participants had physical problems that affected their teaching and make teachers consider a potential career change. To be able to teach, teachers adapted their ways of working according to the challenges brought by age and injuries. The research participants found that the challenges caused by musculoskeletal problems and ageing were an inevitable part of the profession. They emphasised the positive sides of the work: the profession permits varied workdays. In addition, the teachers noted that their work provides them with opportunities to remain physically fit. Teaching health education is a means to lighten the workload of older teachers. PE teachers enjoy their profession and are dedicated to it, despite all the challenges. The interviewed participants clearly experienced work engagement. Our development proposal for teacher education is that future PE teachers be informed about the risks involved in the profession. Such activity helps young teachers reflect proactively on the measures taken to maintain their functioning during their career and on perspectives related to the ways of working.

## 1. Introduction

The labor force is ageing rapidly in most developed countries, so the working careers of older employees should be extended and developed [1]. By the year 2060, 30% of the population in Europe will be over 65 years of age [2]. Finland’s population is ageing rapidly between 2010 and 2030, and the country’s working-age population will decrease by more than 200,000 people by 2050. The data on older teachers show that an increasing part of the profession comprises older employees [3]. Over the past 10 years, the share of over 50-year-olds has grown from 35% to over 40%. The growing teacher turnover and future lack of qualified teachers are sources of worry also internationally [4,5,6,7]. 

To ensure the supply of qualified teachers in the future, the retention of older teachers is of primary importance. While teaching profession includes a lot of similar aspects despite the different roles of class teacher or subject teacher, for example in the physical education (PE) teacher profession there are lots of unique aspects that differs from other teaching professions. These include for example functional nature of the subject, with visible teaching environments. Likewise, teaching in different weather conditions and constant change of teaching environments requires PE teacher to move several times during the day [8]. Older PE teachers must be provided with an individual development path that enhances competence and physical functioning as well as supports working capacity throughout their career. However, it must be taken into account that individual differences grow more clearly along with age: in practice, ageing is a series of physical, psychological and social changes in growth and development, which are influenced by individual choices [9]. These challenges concern all teachers. In addition, they may have subject-specific stress factors, which should be evaluated through their job descriptions. Furthermore, it should keep in mind that PE teachers with higher burnout risk are older than those PE teachers with higher tolerance for stress [8]. 

A challenge for PE teachers is the nature of their profession as physically demanding indoor and outdoor work, as well as the dynamic job description due to changes in pedagogical, conceptual and curricular changes [8]. Finnish PE teachers’ job description is multifaceted in other ways as well. When compared to other teachers, in PE teacher’s job there are special characteristics that are present only in PE teacher’s job. These include, for example, reservation of different facilities and moving between them, transporting equipment, cooperation with sports clubs and organizing sports days, competitions, and equipment stores [8]. The physical nature of the work requires that PE teachers adapt their ways of working according to their coping and functional capacity. Physical work strain is generated by continuous burdening of the circular system through physical activity, the lifting of burdens (moving and lifting sports equipment), and static muscular work, such as assisting with pupils’ movements [10] (pp. 26–27). Additionally, there is high noise exposure in the PE teacher’s profession [11]. To ensure their ability to work, PE teachers must take care of their physical and mental wellbeing [12]. According to various international studies, PE teachers suffer from different musculoskeletal problems, which is also a challenge to their work ability [13,14,15,16,17,18,19]. Of Finnish PE teachers, 31% had at least one disease that reduced their work ability, and 71% of these teachers reported that the work ability issues were related to musculoskeletal disorders [20]. 

In Finland, all PE teachers complete a master’s degree in sport sciences to become a qualified PE subject teacher [21]. In practice, Finnish PE teachers’ work is very independent [22]. Teachers have a lot of autonomy and no external evaluations or standards to meet. National core curricula provide a framework for their teaching. At Finnish comprehensive schools and general upper secondary schools, the teaching duties for PE teacher is 24 h per week. In comparison, for Finnish language teacher the teaching duties is 18 h and for music teacher 21 h per week. National Core Curriculum for Basic Education [23] reformed PE teaching, since the main focus changed to fundamental motor skills. When compared to earlier ones (e.g., National Core Curriculum for Basic Education, 2004), there were no more certain sports mentioned, (e.g., ball games, winter sports, orienteering, track & field, swimming, and gymnastics) as previously. Additionally, with the changes in contents, also assessment has been changed and level of physical fitness does not influence on PE grade [23]. In addition to psychomotor outcomes, there are also social and affective outcomes in the curriculum, but these has not changed in the curriculum reform. 

In this interview research, we examined the challenges to physical and psychological functioning reported by older PE teachers as well as their responses to these challenges. We also discuss whether the factors affecting physical work ability have had an impact on their career and possible career change plans or how to cope with the challenges in their work. After identifying the faced challenges, we try to increase the understanding, why PE teachers have remained in the profession even though they have had some challenges in their profession.

### 1.1. Theoretical Framework 

#### 1.1.1. Ageing and Wellbeing at Work

Wellbeing at work is highly important for the coping and job retention of older employees [24]. Wellbeing at work is a broad concept consisting of multi-level factors—and in practice difficult to define [24,25]. It is affected by factors related to, for example, workplace, working environment, profession, health, employer, population, and socioeconomic position. Wellbeing at work has both individual and organizational effects [25]. It has previously been analyzed from the perspective of health and wellbeing impacts, but the concept has been extended to concern the content of work, workload stress, burnout, workplace relationships, and management perspectives [26]. 

Age affects our wellbeing at work in various ways, adding health problems and weakening work ability. Age can be defined chronologically, biologically, psychologically, and socially, and all these concepts are part of the ageing process [27]. In working life, chronological age determines, for example, retirement age. With respect to working life, the standard definition of ageing has since the 1990s been that people over 45 years of age are middle-aged and those over 55 are older employees [28,29]. The individual signs of ageing are most clearly visible in the decline of muscular strength, deftness, and reaction times [30]. Even though muscular strength decreases with age, the process can be slowed down by physical activity [31]. From the age of 30, aerobic fitness deteriorates by 5% to 22% in 10 years, but the decline is slower in physically active people [32]. Regular physical activity can maintain the cognitive functions, including memory [33]. Furthermore, memory issues can be taken in account during the PE lessons, including memory games in PE lessons, increasing also cognitive functions among students, but also among PE teachers [19]. Prevention exercises could not only prevent from injuries, but also maintain the skill level of teachers [16]. Older employees’ experience can also optimize, select or compensate for many changes, particularly if the skills declining with age are not crucial in their profession and the work environment is not too stressful [34]. Older employees are important for organizations because they are committed to their work [29]. 40% of employees with chronic conditions feel that the conditions complicate their work [28]. Among the 55- to 64-year-old working population, 50% of men and 60% of women had chronic conditions. Chronic musculoskeletal problems clearly increase with age, which must be considered when examining an individual’s ageing and its negative consequences for work. It is still unavoidable that health conditions increase with age and that ageing increases the probability of various occupational health problems [16], which means that an individual’s work ability declines with age [35]. In Finland, the retirement age has been set to 63–65 years depending on the type of job. However, for younger generations the retirement age will be raised. 

An individual perspective on wellbeing at work is the starting point for this study. It is the subjective state of wellbeing experienced by an individual, and this state is affected by the work, management, work environment, work ability, health, personality, and individual reactions [26]. The experience of wellbeing at work is not stable: if the factors determining it change, the experience may change as well. Wellbeing at work and job satisfaction are also influenced by other life situations and overall wellbeing, and they are also connected to our permanent reactions through our personality [26].

Individual wellbeing at work consists of physical, psychological, social, and mental harmony [36,37]. In the “work ability house model” by Ilmarinen [35], the core of work ability consists of health, functional capacities (social, physical, and psychological), family, and social networks. The development of individual wellbeing at work calls for work ability. The nature of work is related to work ability; manual labor weakens our work ability more than administrative tasks do [38]. According to Eriksson [39], the meaningfulness of work is the most significant factor that promotes coping and wellbeing at work. It makes us enjoy our work and commit to it, as well as protects against stress and supports the balance between work and private life. It is essential to feel that your work is meaningful because it increases job satisfaction [37]. It should acknowledge that teachers’ careers are not similar and teacher career can be divided to several phases as Fessler and Christensen [40] have identified. Based on their model, teachers face preservice and induction phases in the beginning of their career. These are followed by competency building and enthusiastic and growing phases. After these, there might be career frustration phase before the career stability and career wind-down phases [40]. Teachers nearing the retirement are in career wind-down phase. Depending on their experiences, they may be satisfied if their career has been rewarding, while they may have also negative emotions, if their career has been unfulfilling [40]. Henninger [41] has created definitions for teachers who had stayed in the profession, but for two reasons, others who are committed to work and still enthusiastic after years of service and still believed they can make a difference are referred as “lifers”, while teachers, who have lost their commitment and enthusiasm for teaching, as troupers. 

#### 1.1.2. Physical Education Teachers’ Work Ability

Research has shown that health issues also pose challenges to PE teachers’ work ability [13,14,15,16,17,18,19]. However, Hüseyin and Bijen [15] observed that very few PE teachers had serious health problems and that their problems were mainly related to musculoskeletal disorders and respiratory diseases. Bizet et al. [42] observed that experienced PE teachers also suffer from chronic injuries in later career stages. The growing work strain of older staff increases the probability of musculoskeletal problems, and especially chronic injuries are more common among older teachers [16]. 

PE teachers are physically more active in their work as well as in leisure time than is the population on average [43]. In practice, older PE teachers’ work ability can be enhanced by the fact that their physical fitness is usually better than average. Furthermore, PE teachers are usually healthier than the rest of the population, a result possibly of their lifestyles, which are mainly healthier than average [16,18,43,44]. As a whole, PE teachers feel well in their work even though ageing poses challenges to their coping [15,20,45,46,47,48]. However, it should keep in mind that there are stress factors in PE teacher profession, e.g., inadequate facilities/equipment, low status of the subject, discipline problems, and curriculum worries [11]. Additionally, hearing problems are quite common among older PE teachers [17].

#### 1.1.3. Ageing Poses Challenges to PE Teachers’ Work Ability as Well

Wellbeing at work affects considerations of whether or not to continue one’s working career [49]. Sustainable work ability throughout one’s career requires that one’s health also lasts throughout the career [50]. An individual’s physical fitness declines and recovery slows with age, which weakens the sense of resource balance [39]. Teachers’ functioning is determined by their mental and physical capacities, which promote their coping with professional requirements [50]. Problems arise from PE teachers’ physical problems near the end of their career [42]. Individual PE teachers’ views on work ability vary and depend on the cultural context as well as the working environment and conditions of work [46]. Additionally, aging arises in physical constraints, e.g., higher noise levels and serious voice disorders [11]. 

Hüseyin and Bijen [15] observed in their study that PE teachers’ physical fitness and health, mental health, stress tolerance, work ability, work motivation, work atmosphere, expertise, and management at the workplace were at a high level. This view is supported by the observations of Mäkelä and Hirvensalo [20] that PE teachers’ experience of their work ability is good. When compared to other teachers, Vedovato and Monteiro [51] have found that 42.6% of teachers in Brazil had good work ability, and 35.3% of teachers reporting moderate or low work ability. Similarly, Vangelova et al. 45 reported that most of the teachers had good (44.8%) or excellent work ability (15.8%) in Bulgaria. However, it must be considered that older PE teachers have more challenges related to work ability than do their younger colleagues [15,52]. For example, younger PE teachers have reported higher level of work ability, physical fitness, mental health, and physical health than older PE teachers. However, in stress tolerance there were no differences between age groups [15]. 

## 2. Materials and Methods

Based on earlier study project PE teachers’ careers, job satisfaction and career intentions [8] related to job satisfaction, career intentions and turnover of PE teachers in Finland, a sample of older PE teachers were selected for a closer inspection. We wanted to have both men and women participants from different locations from Finland. We contacted PE teachers based on the previously collected data. From this sample, eventually six Finnish PE teachers were interviewed in the study. Inclusion criteria were age over 55 years old, and teaching career had lasted for more than 30 years. We chose PE teachers with a long working career, which enabled them to give in-depth descriptions of their experiences at various phases of the career. The interviewees included three women and three men, based on random distribution. Five of the participants had graduated as PE teachers from the University of Jyväskylä’s Department of Physical Education in the 1970s, while one of them had completed the Master of Sport Sciences degree in the mid-1980s. 

The aim was to find out how reduced physical work ability and different musculoskeletal problems had affected their work and whether these had made them consider career change. The teachers selected for the study reported being satisfied with their work. They were contacted by email and invited to participate in the study. Analyzing satisfied teachers allowed us to identify strategies that can be used to support coping at work [53]. All subjects gave their informed consent for inclusion before they participated in the study. The study was conducted in accordance with the Declaration of Helsinki, and the protocol was approved by the Ethics Committee of the University. 

Qualitative methods were used in the collection, transcription, and analysis of the research data [54,55]. The qualitative analysis consisted of a series of interpretations that visualized the world described by the interviewees [54] (pp. 3–4). Through the researchers’ phenomenological and hermeneutic view on humans, the experiences highlighted by the interviewees’ narratives, as well as their understanding, could be made meaningful when interpreting the things said and making conclusions based on them [56]. The hermeneutic perspective was influential in the phase where activities and things said were interpreted. In practice, the first author of this article created an overall picture of the data, analyzed what was said, and reported the results thereof [57]. 

The interviews were carried out as thematic interviews [58] in the place chosen by the interviewees, in Finnish. The interviews lasted for 60 to 120 min, and their recordings provided 90 pages of transcribed data. The thematic interviews provided the participants with opportunities to share their experiences extensively [59]. The researcher asked them to specify questions related to earlier research findings [8]. In thematizing the interviews, we also considered themes relevant to literature on the topic [38,40,60,61,62], such as wellbeing at work and its determinants. The aim of the interviews was to clarify, for example, how the teachers had maintained their physical fitness and functioning, and whether they had had problems related to physical functioning that had affected their work as PE teachers. In addition, the aim was to find out if they had had work- and exercise-related accidents, physical restrictions or chronic injuries and changes in the work that may have increased the stress-levels that affect coping at work and potentially make them consider a career change. Research questions included main questions, e.g., “What are the main reasons for continuing in the profession that increases your well-being?” or “How is your health at the moment?” In addition, interviews involved subquestions about the profession, e.g., “What kind of challenges your health has influenced on your job?” Questions were constructed with the knowledge from previous study [8] and validity of the questions were verified by two experienced PE teachers (Supplementary Materials). 

The transcription started by listening to the interviews, so that the researcher could better perceive the message of the interviewees. In the first analysis phase, the data were broken down into as small subgroups as possible. A word processing program was used to highlight each analyzed subarea in different colors [63]. Based on earlier research (e.g., [8,38,40]), theory-driven content analysis was used to analyze the data. Using available studies or theories, the researcher first identified the key variables or concepts and used them as a primary coded classifications, then operational definitions for each category were determined using previous studies. From previous studies, physical functional challenges, coping and career change plans were raised to this study (e.g., [8,18,38,40]). Semantic contents were identified in the texts and divided into predefined categories, but new categories (PE teacher’s inspiring work, changing work) was also created in this phase. Categories that were close to each other were then combined. Eventually, five categories were identified: (1) Physical functioning challenges, (2) PE teacher’s inspiring work, (3) Changing work, (4) Age affects coping and, (5) Career change plans. New categories were based on considerations that these mentions were commonly mentioned and did not fit quite clearly to other categories. The hermeneutic circle was used to understand themes arising from the data so that we sometimes returned to the preunderstanding arising from literature and earlier research, against which we reflected the understanding obtained based on the data [64]. The interpretations were validated and compared to the theoretical framework, based on which conclusions were made about the participants’ message and my observations. A description of the participant’s demography is presented in Table 1.

## 3. Results

Being an experienced PE teacher yielded long stories about the experiences, challenges and changes that have occurred. Hereby, we account the for the findings in five headings structured line and around with the research questions: (a) challenges in physical functioning, (b) inspiring work, (c) changing nature of PE teachers’ work, (d) how age affects coping and (e) career change plans. 

### 3.1. What Do Older PE Teachers Say about Their Physical Functioning Challenges? 

Several international studies have reported on different knee, shoulder, and back problems [16,20,65], similar to the interviewees of this study: “*Joint and knee pains are something that sometimes make me worry*” (Tanja). Injuries caused worries about coping, but the teachers still said their physical functioning was good: “I myself think I’m rather fit because I don’t have any physical problems, despite a few small incidents that I’ve had” (Jouko). Immediately thereafter the teacher stated: *“You see, I had one accident when we were on that artificial ice, it was pretty cold and there were cracks on the ice, so my skate hit one and my knee twisted, and the meniscus tore”* (Jouko). The teachers had had knee surgeries after accidents that occurred at work. An interviewee shared the following story:

*Twenty years ago, I had an accident, so I had an operation on my knee, but it’s quite all right now; it was an accident at work that happened when we were having “sack fights” on the beam with upper secondary school boys and one of them lost his balance and hit my knee.* (Pertti)

All the interviewed PE teachers had musculoskeletal problems, some resulting from work and others from earlier sports hobbies. Older PE teachers described these problems illustratively: “*At this age, it’s clear that you get musculoskeletal challenges, and they certainly restrict what you can do. For example, you can’t handstand with wrists like mine… osteoarthritis and something in the knees…*” (Minna). Another teacher described his physical problems as follows: “*Though, especially last year, my left knee caused me worries, it was swollen nearly all winter and showed symptoms all the time…*” (Tanja). One teacher explained: *“I’ve had a couple of knee operations, now I have nothing; at the moment there’s just a small tear on my shoulder, but we’re pondering whether to operate or let it be as I can cope with it”* (Jouko). Despite their musculoskeletal problems, all the interviewees were willing to continue working as PE teachers. Since PE teachers are also in charge of students’ safety in lessons, they have to consider, whether these challenges may endanger the safety of students. Hence, it may be considered that despite the challenges, they were able to continue in PE teaching profession. They did not find that the problems would affect teaching enough to cause a significant challenge. They also identified various means that helped them continue teaching despite the musculoskeletal problems. One important means was that they need to remain physically fit. The significance of warming-up was highlighted as a means to prevent injuries especially before demonstrating movements or joining activities that require sudden exertions: “*You need to keep fit and, of course, think what you can do, and if you have warmed up… so that you don’t join some extreme activities out of the blue*” (Jouko). Musculoskeletal problems made the interviewees worry about their possibilities to carry out teaching. 

### 3.2. PE Teacher’s Work Is Inspiring and Enables the Maintenance of Work Ability 

The teachers in this study found that teaching PE and participation in pupils’ activities was important for staying fit. *“...I also participate with them, in different situations, well, sometimes I take part in their games and, like I said, I always skate and downhill ski”* (Tuiti). Another teacher emphasized *“being in motion all the time during lessons, in extremely active motion with the pupils”* (Pertti). Work can be a major source of wellbeing for an older employee. This was echoed in several comments, as expressed by one of the interviewees: *“Of course, the fact that you sometimes get to go to sunny snowfields and can spend a lot of time outdoors adds freedom to our work”* (Heikki). 

The age awareness of the PE teachers in this study and the significance of active maintenance of physical functioning were important reflection topics. They had taken into account that “staying physically fit feels more and more important each and every year” (Jouko). Another interviewee emphasized that “*when approaching sixty, most of us certainly start having even physical challenges*” (Heikki). The participants highlighted that ageing also makes you accept yourself the way you are, which also helps you accept more challenging teaching situations calmly and without offending, for example:

*I wanted to say about ageing and teacher identity that perhaps it gives you a kind of commonplace self-acceptance, so that when you have conflict situations with pupils or when someone misbehaves, your attitude will be that well, you’re like that and I’m like this, and from this starting point we should go on together.* (Tuiti)

With respect to the maintenance of mental functioning and recuperation, different hobbies, and support from one’s family have promoted coping: “*I have had the chance to do hobbies and attend courses and such; exactly this family support has allowed me to do things*” (Heikki). PE teachers’ leisure hobbies were oriented to different physical activities, but their main idea was relaxation and mental recovery rather than target-oriented development of physical functioning: *“In any case, physical activity is significant for maintaining my work ability, and so is seeing my friends, so maybe one should do even more other things than exercise”* (Tanja). Hobbies helped one recover from work and support work ability. All the participants engaged actively in free-time hobbies, which mainly comprised physical activities such as the following: *“Hobbies (floorball) mainly mean relaxation, self-realization, and when you distance yourself from work through them, you will clearly have more energy at work. Anyway, I live a really healthy life during the week, and also otherwise”* (Pertti). It seemed that alongside the maintain physical work ability, having social contacts in hobbies helped mentally to get rid of thoughts from the work. In addition to sports, the interviewees engaged in other types of activities in their free time, and one of them formulated it as follows: “*I have awfully many hobbies, and politics, and so on*” (Tanja). One interviewee stated that *“there’s this language circle, which is something totally different”* (Tanja). Another interviewee went to open-air dancing at summer weekends and emphasizes how relaxing it is: “*I go dancing really actively, simple ballroom dance stuff”* (Pertti). While women mentioned that they maintained their work ability with body maintenance, e.g., stretching, pilates and gymnastics, men practiced more different ball games. One participant also mentioned that she participated to one course, which helped her to maintain work ability. *“In 2009 I graduated as a Feldenkreis instructor. With the Feldenkreis-method, I take care of my own kinesthetic sense and it helps if there is anything stuck.*” (Tuiti).

### 3.3. Teacher’s Work Is Changing—Does It Promote Coping?

Teacher’s work has always undergone changes. According to the interviewed teachers, the recent development has significantly broadened their job description as, for example, health education has become an independent subject and physical qualities and the maintenance of functioning have replaced the various sports. Health education as a separate subject has increased the need for qualified HPE teachers. At the same time, some other teachers are also teaching health education (e.g., biology or home economics teachers). The change was described as follows: *“We older teachers just wonder why they don’t mention any sports and it’s all about some “observing” and “sliding on the snow” but not skiing”* (Tanja). As the National Core Curriculum for Basic Education [23] highlights the fundamental motor skills as a core of the teaching, it has removed the certain sports from the curriculum. Hence, some teachers have found it awkward that there are no sports anymore in the curriculum. However, this has not changed the fact that PE teachers can teach kicking as a part of a football lesson. 

According to the National Core Curriculum for Basic Education [23] teaching aims at a working culture that supports learning, participation, wellbeing, and a sustainable way of life. Instruction in PE must also promote the maintenance of physical, social, and psychological functioning. The learning environment must promote pupils’ activeness, self-directedness, and creativity. The interviewees explained that earlier teachers, in practice, could decide on all teaching content, in other words, what was done, but now the core curriculum encourages them to involve pupils in the planning of teaching. One of the teachers described that teacher used to be authority and decide what was done, but now teaching is planned together with pupils: *“Perhaps teachers had more authority and could decide what to do, but now we also must try to listen to pupils, that’s a big change”* (Tanja). The curriculum changes have made teachers reconsider their own pedagogical methods: *“It certainly makes you wonder if we still can change the teaching methods in such a way that would better share the joy of sports, I don’t know”* (Tanja). The changes are stressful, but *“with age one has learned to tolerate change and the stress it causes”* (Minna).

*Some of the interviewees found that the change in teacher’s work turns teaching into the organization of physical activity situations, which actually adds positive variety to their work. One of the participants states: “Perhaps the change in teacher’s role so that we become organizers of situations, which I have actually liked...such organizing rather than that old teacher-centered approach that naturally first prevailed”* (Heikki).

The interviewed PE teachers had been assigned completely new tasks at later career stages, which changed their job description. One significant change was instruction in health education. Some of the teachers find that it brings variety and is an opportunity to make work lighter if one’s mobility declines: *“It can somehow change your work if you feel your mobility is clearly declining; so we’ve talked in these development discussions that one can then increase the amount of health education and change the content of work somehow, if needed”* (Tanja). Similar findings have been found also from other countries, as coaching alongside teaching has brought some alternation for the PE teaching profession for some teachers [66]. At the same time, the teachers experienced that teaching two different subjects was challenging. Moving from PE venues to the classroom was also exhausting: *“Teaching health education is difficult because when returning from the ice rink or playing fields you have perhaps 10 min to change clothes and prepare before entering classrooms; that is currently perhaps the most stressful thing in schoolwork”* (Jouko). 

### 3.4. Age Affects Coping—Activities Are to Be Adjusted Accordingly 

The participants identified some ageing-related challenges to their teaching, and one of the ways to respond to the challenges was to adjust teaching according to their work ability. The interviewed teachers have had opportunities to influence the content of their work, as one of them states: *“I’ve also had a chance to influence my working duties”* (Minna). Essential in individual work adjustment is that it is done within the framework of the job description. In practice, it means that specific parts of one’s working methods and individual tasks are adjusted [67]. Older teachers are relatively independent in their teaching and its adjustment: *“You can do things in the way you think fit without needing to check on everything; I mean you can use your common sense every now and then”* (Minna). In later career stages, the teachers adjusted their teaching so that it is physically less exhausting than it was earlier: *“I use students or videos, or they (students) figure it out themselves…”* (Minna). The participants emphasized that the possibility to plan one’s own work and set its rhythm has affected their job satisfaction. Older teachers aimed at making teaching meaningful for themselves and for their pupils, as one of them explains: *“I keep on thinking how I can make my own work as easy as possible, so that I personally enjoy it, but still taking care that the tasks remain diverse, and pupils enjoy themselves as well”* (Tuiti). The participants said that as one gets older, physical work needs to be balanced with one’s functional capacity. They were aware of their own limitations and describe the balancing as follows: *“Balancing every now and then; over the life course you’ve had to understand that sometimes you need to calm down a bit, you can’t do it all at full speed”* (Jouko). Another teacher underlined the issue by stating: *“There has to be some sense as I’m this old”* (Tuiti). 

Age also brought challenges to the teaching of sports techniques and particularly to demonstrating movements and exercises. One participant explains: *“I’ve asked them to forgive me a little for my technique because I’m already this old”* (Tuiti). The teachers have understood that they need not do everything themselves but can have skilled students demonstrate the movements. The problem can sometimes be that the teacher becomes inspired to join the activities even after having decided to be careful: *“Then I notice only later… when we play some ball game, that I may be inspired to join”* (Minna). Age also caused insecurity, pressure, and reflection on how one looks when teaching new sports that are popular among adolescents. One of the teachers had considered introducing Zumba in class but had then changed his mind: *“But then I thought pupils would laugh at me and gave myself permission to not do it”* (Tanja). 

The interviewed teachers recognized the strains of their work and are aware that continuous standing is more exhausting for them than it is for young teachers, as are bad working positions (ergonomics, e.g., assisting students’ movements) and moving around equipment:

*Anyway, in that work you stand on your feet all the time, which is great and good, but it probably starts to strain your joints when you’re in bad positions and have equipment to carry, and that should perhaps receive a bit more attention.* (Heikki)

Some of the teachers highlighted that their working days sometimes include long periods of physical strain. Their long experience enabled them to address the issue with their supervisors in order to change the activities:

*Last year I had eight hours of PE a day, so I also skated eight hours a day. That’s something I’ve talked about with the principal, that this could be forgotten in the future, nobody needs it—then you sit all evening under a blanket to get back to your basic temperature. Because if you happen to work up a sweat, soon you’ll be cold for hours.* (Tuiti)

PE teachers’ own body is their main tool, so they must understand that the work is physically strenuous, and that recovery requires regular body maintenance [12]. Recovery plays an important role. According to the interviewees, their possibilities for recovery had increased when their own children had become independent and they no longer needed to engage in activities all evening: *“There’s more time for recovery because you’re no longer in the family, you can rest enough after work, as long as you have the patience to do so”* (Tanja). Another teacher also described the ways of recovery from work as follows: *“I lead a basic, simple life. I exercise and eat rather healthy, sleep well—and that’s such an important part of my recovery”* (Tuiti). 

### 3.5. Career Change Plans 

At different career phases, the interviewed PE teachers had also considered career exit. One older teacher explained: *“I graduated at the age of 25 and have worked for nearly 40 years, and then I think there’s probably also other stuff in the world I want to do”* (Tuiti). Different injuries caused insecurity about the ability to continue in the profession:

*In the eighties I underwent meniscus removal… moving was difficult, so I started to think how I could do it for the rest of my career as I could no longer jump on apparatus or squat jump; I namely had this osteoarthritis and now an artificial joint.* (Heikki)

The teacher had worked as a PE teacher for more than 30 years but had to change to occupational safety tasks in the last years of his career. The following interviewee was also worried about the possibility to continue until retirement age:

*Once when I worried about my ability to continue in this work, I had the opinion that if I lose my work ability after a long career in teaching, I should find some other profession through a relatively short education, in a way a new life.* (Pertti)

The teachers had considered retraining when their work ability had declined. However, they had not undertaken career change because they found their work was meaningful. The interviewed PE teachers mentioned that their chosen physically active profession afforded the possibility to be physically active in their work which motivates them and keeps going. When one’s work is meaningful, it inspires and motivates: *“In fact I like physical activity so that it’s not unpleasant at all to go and do it, and often I think I’m privileged because I can do it at work—there are not many professions where that’s the case”* (Tanja). One of the interviewees crystallized the significance of the profession as follows: “*This is actually not work but a way of life. Being with young people, in a way you get to fulfil yourself*” (Pertti). Teachers enjoyed their career choice also in older age: “*Not even once have I thought about wanting to change... on the contrary, year by year I’m happier about a job like this*” (Minna). One teacher had a break from teaching PE as she was working as a class teacher. She even decided to change completely class teaching position, but eventually she decided to stay on PE teaching (Tanja). These findings highlight the Fessler and Christensen [40] career cycle and the last phase of the cycle, career wind-down. Teachers in this study have pointed out their happiness about their career choice and how they felt their career rewarding since they have had an opportunity to make a difference with children and influence their lives. While good working ability has helped PE teachers to stay in profession, it must keep in mind that there must be also supportive environment to stay in the profession thorough the career, including personal and organizational elements. These include for example family and positive critical incidents (family) and societal expectations, public trust, and management style (organizational) [66]. 

## 4. Discussion

This study describes older PE teachers’ experiences of challenges to their physical and mental functioning during their career as well as how they responded to these challenges. The participants reported mainly about different physical problems that affected their work. They adapted to the requirements of their work ability and compensated for their declining mobility by, for example, warming up before demonstrating and by having pupils help in certain tasks. The possibility to influence one’s working methods and arrangements was a prerequisite for physically and mentally meaningful work. The study provided parallel results with an earlier study [42], where physical problems were not observed to be a significant factor in determining older teachers’ career change or retirement plans. In this study, similar to the earlier studies [42] the older PE teachers explained that physical restrictions and chronic injuries still made them reflect on a career change. However, while the study by Bizet et al. [42] Concentrated on work ability of PE teachers, this study also studied the coping methods to deal with the challenges for PE teachers. While PE teaching includes several common aspects despite the cultural context (e.g., helping students, showing movements, assessment, etc.), there are differences in educational systems (e.g., time allocation and actual implementation), teaching environments (facilities and equipment), subject and/or teacher status, curriculum emphasis (e.g., sports-oriented or physical activity & wellbeing) [68], job description (teaching multiple subjects or working as a coach) [69,70] which may generate differences between different educational settings. 

The teachers’ musculoskeletal injuries affected teaching, restricted activities, and occasionally prevented active physical participation in class. The interviewees found that the challenges caused by musculoskeletal problems and ageing were an unavoidable part of the profession. While teachers found that musculoskeletal problems made them worry about their future, they all had managed to deal with the profession and carry-on teaching. However, this should be understood also in physical education teacher education and consider what are the career opportunities if the job strain is too much with the musculoskeletal problems. On the other hand, it may be much of coincidence. If there is an opportunity to change your job to other than PE teaching, it may be that the teacher changes his/her job. However, if there is not an opportunity, then he/she will continue to teach PE. 

The interviewed teachers emphasized the importance of sustaining physical fitness as one gets older, as nearly all of them already had physical challenges. Physical fitness unavoidably declines along with ageing, and if physical strain simultaneously increases, the risk of various health problems grows. The increased strain at later stages of their career when compared with that of other teachers is the result of PE teachers’ work being more challenging than other teachers’ work because they must be physically active in PE lessons [42,71]. 

One of the factors that promote PE teachers’ commitment to work is the possibility to be active and work outdoors [42]. The profession allows varied working days and activities such as skiing with pupils on sunny snowfields in the spring. The teachers also noted that their work provides the opportunity to remain physically fit, better fit than referents [18]. In addition, older teachers participated in PE lessons, though more carefully than they had earlier in their careers. 

The changes provided by the National Core Curriculum for Basic Education [23] have altered the nature of Finnish teachers’ work. Pupils are now expected to participate actively in planning and teaching activities. Pupils’ participation at all stages of teaching has made the interviewed teachers’ role more guidance-oriented and simultaneously eased their workload. Teachers highlighted in an earlier study of this research project [22] that pupil-centered approaches and the opportunity to influence one’s own working methods increased their wellbeing at work. 

The teaching of health education and increased administrative tasks provided significant changes to PE teachers’ work. In this study, we observed that, for some teachers, these changes increased the pressure of work and the sense of haste, particularly because returning from sports facilities took time and left insufficient possibilities to prepare for the next lesson. According to our observations, the participants’ mental wellbeing was supported by their increased free time, which enabled better recovery from work. This was related to such factors as one’s own children no longer needing care and attention. In addition to recuperative physical activities, free time was spent in various other hobbies, such as politics, language studies, and social dancing. Diverse hobbies brought balance to life and supported coping at work. 

In this study, like in the earlier studies [42,72] it was found that the interviewed PE teachers had reflected on the possibility of finding other work, but they had not started another education or even seriously considered another profession. One reason they mentioned was that getting another degree at their age would be too long a process. However, the main reason was that PE teaching was the profession in which they had always wanted to work. They enjoyed their work and were committed to it. In practice, work is something that promotes their wellbeing. Work that fosters the experience of meaningfulness also supports coping and wellbeing [39]. The PE teachers worked in a profession that they enjoyed and in which they wanted to continue, despite the challenges. They clearly experienced work engagement. However, it might be that they have been able to influence their job more than PE teachers normally, and this has helped them to remain in the profession, as it has been previously found [66]. 

The PE teachers in this study felt that their work was a source of wellbeing. They were enthusiastic about their job and wanted to continue until retirement, despite the physical challenges. Because of their work ability challenges, they reflected on how to continue in their beloved profession. In contrast, in a study conducted with PE teachers in Quebec, only a few felt they would be able to continue actively in their profession until retirement age [42]. In another study in Australia, experienced PE teachers felt that they have reached their “use-by-date”. However, instead of work ability, lack of respect and opportunities for decision-making were the main reasons to consider leaving [73]. This study showed that the increase in health education added variety to PE teachers’ work and reduced their workload. Supervisors should indeed consider giving older teachers opportunities to teach more health education, which would lighten their physical workload. However, classroom teaching must be scheduled so that teachers have sufficient time for preparation between PE lessons. Additionally, age management would help to diminish the workload. For Older PE teachers planning the days to two parts would minimize the transitions when the morning lessons could be PE and afternoon lessons health education.

It is highly important to find ways to support the work ability of PE teachers. This study indicated that older teachers with high wellbeing have various means to solve physical functioning challenges by adjusting their work. It was also a significant observation that, despite their challenges, the teachers were enthusiastic about their work and found that their wellbeing at work was of a high level. With the ageing of the labor force, employers should understand the challenges involved in PE teachers’ work so that they can ensure employee wellbeing, work ability, and work retention. The preservation and promotion of work ability enable wellbeing at work, which is the key to long careers [74]. We should also remember that instruction by satisfied teachers is of a high quality [75] Recently, in Finland tailored rehabilitation courses have been started for PE teachers. In these courses maintaining the work ability is one main purpose, but also empowerment by others is one key purposes of the course that can help to continue in the profession. Experiences from this research can be used in the rehabilitation courses and support the long careers of PE teachers also in the future. Likewise, more focus should be directed also to preservice and in-service training of PE teachers. For preservice teachers, courses that are related to wellbeing and work ability in PE teacher profession should be included in the physical education teacher education and highlight the threats that can be harmful and cause impairments. 

Teacher education should also provide information on the risks related to the profession of PE teacher [17]. This helps young teachers reflect proactively on the measures taken to maintain functioning during their career as well as on perspectives related to the ways of working. Moreover, better understanding of the changes to functional capacity brought by ageing provides teachers with the opportunity to address the issue in development discussions during their career. 

An ethics perspective has guided our entire research process. At the reporting phase, we have ensured the participants’ anonymity and confidentiality related to their city, school, and all other personal information. It must be noted that the results of this study cannot be generalized to all older PE teachers, but the results are probably similar among older PE teachers who are satisfied with their work. All the teachers who participated in the study had a long career behind them, which can be regarded as a strength of the study. Despite age-related decline of physical functioning and different musculoskeletal problems, they had managed to continue in their profession. They also felt their wellbeing at work was optimal and presented concrete means of individual work adjustment. However, it should keep in mind that musculoskeletal problems are not the only threat for work ability. Since the working environment is often very noisy, there might be also concerns with hearing or voice problems [11]. 

This kind of research is warranted to better understand PE teachers’ careers and professional lives. It is important to learn why some PE teachers are capable to continue in the professional while others feel the profession too exhaustive and exit the profession. In addition, experience of PE teachers should also be used in organizational socialization. Experienced PE teachers could work as mentors for younger generations and via that way, gain also extra appreciation. Low status and marginalization are commonly reported in PE teacher profession [73]. Likewise, professional development programs for PE teachers in certain age could help PE teachers to share their concerns with others at same age and probably find solutions with other colleagues to sustain in the profession even though there might be some musculoskeletal or other problems. 

## 5. Conclusions

Older PE teachers seemed to be quite satisfied to their jobs. However, certain coping skills is needed to compensate the declining level of skills and ability to demonstrate movements. This may also mean different selection of sports included in the physical education or optimizing the workload during the day. Certain age management should also take in account and promote the possibility to reduce PE teaching and increase health education teaching. Additionally, days could be splitted to two parts, in the morning PE and in the afternoon health education. This would reduce the workload as the transitions during the day is limited. In the future additional modes to promote the work ability should be considered., e.g., taking the workload issues as a one topic in the initial teacher training but also consider PE teachers professional development that promotes work ability after middle age. This study increased our understanding about the PE teachers work in their career wind-down phase and how they cope with the challenges. Future research could be conducted to PE teachers in their fifties, who decide to eventually leave the profession and compare to teachers who decide to stay. What are the reasons why some teachers quit, and some keeps going. 

## Figures and Tables

**Table 1:** General demographics of the sample.

Participant (Pseudonym)	Gender	Age	Teaching Experience	Class Degree
Tanja	Female	59	33	Upper- and lower secondary
Heikki	Male	60	36	Upper- and lower secondary
Pertti	Male	58	32	Upper- and lower secondary
Jouko	Male	59	35	Upper- and lower secondary
Tuiti	Female	58	32	Upper- and lower secondary
Minna	Female	58	30	Polytechnic

## Data Availability

Not applicable.

## Supplementary Materials

The following supporting information can be downloaded at: https://www.mdpi.com/article/10.3390/ijerph192114250/s1, Interview script.
