# Supplementary material for: Older Physical Education Teachers’ Wellbeing at Work and Its Challenges
Source: Int J Environ Res Public Health. 2022 Oct 31;19(21):14250. doi: 10.3390/ijerph192114250 (PMC9658279; doi:10.3390/ijerph192114250)
Supplement: Supplementary file 1 [file ijerph-19-14250-s001.zip › ijerph-1990122-supplementary.pdf]

Supplementary: Interview script

When did you graduate as a physical education teacher?

How has your career progressed since you started at your first job?

What does work as a physical education teacher mean to you?

Do you enjoy your work, if so, how?

What excites you about teaching?

What are the ways you have developed your skills during your career?

How do you see that the work of a physical education teacher has changed during your long career and what challenges and positive aspects have you experienced?

What have been the most difficult things during your working career and why? How have you survived them?

How do you feel about your health now?

How has the health test affected your work and what possible challenges has it created?

Do you keep up with the students within your physical capacity?

What do you think your work ability will be like in the future?

Evaluate whether you can survive in your job at the same level in another two years?

Has the feeling of physical load at work increased significantly as you got older and how does it show up in your daily work, if it has increased.

What is the situation at the moment, are there any challenges at work?

Does the work of a physical education teacher cause problems?

If applicable:
Kerro vammoista tai rajoitteista, ovatko ne tulleet liikunnanopettajan työssä?

Have the operations affected teaching?

Continues to all:

What kind of physical condition do you think you are in?

How have you taken care of your work condition?

Has maintaining your fitness brought challenges?

What kind of hobbies have you had?

New hobbies such as skateboarding have increased among young people. Have you had to learn and practice them?

Did physical education teacher training provide sufficient skills in the initial phase? If not, what should have been more?

Does the physical education teacher need further training?

What have been the most difficult years during your working career?

Are you able and do you want to work as a physical education teacher until retirement age?
